# Cardiovascular Immune‐Related Adverse Events of Immune Checkpoint Inhibitors: A Systematic Review and Meta‐Summary of Case Reports

**DOI:** 10.1002/cnr2.70675

**Published:** 2026-09-17

**Authors:** Shea‐Lee Godin, Tyler Kingma, Ashwin Pillai, Obada Kholoki, Agnes S. Kim

**Affiliations:** ^1^ Department of Medicine University of Connecticut Health Farmington Connecticut USA; ^2^ Department of Medicine, Division of Cardiology University of Connecticut Health Farmington Connecticut USA

## Abstract

**Background:**

Reports of immune checkpoint inhibitor (ICI) therapy‐associated immune‐related adverse events (irAEs) exhibit considerable heterogeneity, particularly when comparing data from clinical trials and real‐world observational studies.

**Methods:**

We conducted a systematic review of real‐world observational studies (case reports and series) reporting cardiovascular irAEs in PubMed and Embase from inception to April 1, 2024. We analyzed 116 case reports from 115 studies and highlighted the commonest irAEs, treatments instituted, and ensuing outcomes.

**Results:**

Myocarditis was the most common, accompanied frequently by pericarditis and heart failure. Treatment typically included corticosteroids and discontinuation of ICI therapy. Higher doses of corticosteroids appeared promising. Refractory cases benefited from plasmapheresis, intravenous immunoglobulin, mycophenolate, tacrolimus, or infliximab. Rechallenging ICI therapy after irAEs resulted in mixed outcomes.

**Conclusions:**

Given the heterogeneity of data, analysis of aggregated data from central repositories like Side Effect Registry Immuno‐Oncology (SERIO) would be invaluable.

## 1 Introduction

Despite significant progress in surgical techniques, chemotherapeutics, and targeted antineoplastic regimens, cancers collectively represent the second‐highest cause of mortality, years of life lost, and disability across the globe [1]. This is particularly concerning given that over two‐thirds of incident cancers are estimated to occur in countries classified as low‐income or middle‐income on the Sociodemographic Index (SDI) [2]. As the collective demographics in these nations age, the cancer burden is likely to increase even further.

The transition from cytotoxic chemotherapy towards personalized cancer treatments using targeted therapy and immunotherapy has contributed to improved survival in many patients with cancer. Immune checkpoint inhibitors (ICI) are among the most promising of these targeted therapies. They act principally by helping T‐cells overcome the immune “exhaustion” induced by a malignancy via various co‐stimulatory signaling molecules on antigen‐presenting cells (APCs) and T‐lymphocytes, including the cytotoxic T‐lymphocyte antigen 4 (CTLA‐4), programmed cell death protein (PD1), and its ligand (PDL1) [3]. ICIs have demonstrated efficacy across a broad spectrum of malignancies. To date, regulatory approvals encompass metastatic melanoma, non‐small cell lung cancer (NSCLC), small cell lung cancer (SCLC), renal cell carcinoma (RCC), cutaneous squamous cell carcinoma, urothelial carcinoma, colorectal cancer with microsatellite instability‐high [MSI‐H] or mismatch repair deficient [dMMR] tumors, hepatocellular carcinoma, Merkel cell carcinoma, gastric and gastroesophageal junction adenocarcinoma, esophageal squamous cell carcinoma, cervical cancer, endometrial carcinoma, and triple‐negative breast cancer [4].

However, despite the promising anticancer efficacy, the use of these ICIs is associated with a unique pattern of adverse reactions christened immune‐related adverse events (irAEs) [5]. irAEs are myriad, capable of affecting virtually all organ systems ranging from the heart to the lungs, liver, intestines, and pituitary [6]. It is estimated that nearly 60%–80% of patients treated with ICI experience some form of irAE [7]. The rate of severe or life‐threatening irAEs varies by the agent used. Severe or life‐threatening irAEs (classified as grade 3 or grade 4 per the Common Terminology Criteria for Adverse Events (CTCAE)) are seen in up to 42% of patients who receive ipilimumab, whereas they are seen in ~15% of patients who receive PD1 inhibitors including pembrolizumab and nivolumab [8, 9].

Prompt recognition of an irAE is imperative to discontinue the offending agent, prevent further damage, and institute effective treatment as expediently as possible to optimize overall clinical outcomes. Given the significant increase in the use of ICI over the past decade, it is increasingly likely that providers across diverse specialties will encounter irAEs. Thus, it behooves all patient‐facing clinicians—physicians, advanced care providers, and clinical pharmacists alike, to familiarize themselves with the more commonly encountered irAEs and to arm themselves with the knowledge of diagnosis and management.

A recently published meta‐analysis by Sharma et al. explored 54 randomized clinical trials and showcased the significant association between ICIs and risk for myocarditis and pericardial disease [10]. However, the study noted discrepant findings from the 24 observational studies they included. The reasons for this discrepancy were likely multifactorial, ranging from differing definitions of adverse events to differing indices of suspicion leading to variable rates of diagnosis. However, since the completion of data extraction by Sharma et al. in 2021, the use of ICIs has doubled, and there has been a concurrent surge in published literature reporting adverse events at multiple centers [11, 12]. Thus, in this updated review, we aimed to collate the most current real‐world observational data reporting the cardiac complications associated with ICI use.

## 2 Methods

Our systematic review was structured and reported in accordance with the Preferred Reporting Items for Systematic Reviews and Meta‐analysis (PRISMA) guidelines.

### 2.1 Data Sources and Searches

We conducted a comprehensive search of the PubMed and Embase databases from inception until April 2024 using the search terms “case report,” “Pembrolizumab,” “Nivolumab,” “Ipilimumab,” and “cardiac.” Eligible studies met the following criteria: (i) case reports and clinical case series, (ii) active treatment with ICI, (iii) laboratory or imaging‐proven cardiovascular irAEs, (iv) documented patient outcomes, (v) written in English. Case reports on cardiovascular irAEs caused by the ICIs pembrolizumab, nivolumab, ipilimumab, or any combination of these agents were reviewed if abstracts and full text were available. The automatic filters applied to the database searches were full text and abstract availability in English. We excluded studies based on the following criteria: conference abstracts, repeat publication, and incomplete reports. The more recently FDA‐approved agents of durvalumab and atezolizumab were also excluded from our analysis, given the relatively infrequent use and rarity of reported adverse events in the literature. Seven reports identifying monotherapy or combination therapy involving new ICI therapy were identified; however, due to the rarity of the reports and the infrequency of their use, they were excluded from our analysis.

During our case search, we excluded the Cochrane Library because it primarily indexes systematic reviews and randomized controlled trials (CENTRAL), which do not align with our inclusion criteria, which are strictly focused on case reports/series. We did not search Springer directly, as it is a publisher database; its relevant clinical journals are indexed in PubMed and Embase, ensuring they are captured in our search. Finally, while Web of Science and Scopus are valuable tools, prior studies suggest that for clinical case reports, the combination of PubMed and Embase provides high sensitivity with minimal overlap, whereas broader databases yield more duplicates and lower yield. We believe our current search strategy provides a representative and comprehensive sample of the clinical phenotype of cardiovascular irAEs.

Two authors (SLG and TK) independently and manually reviewed all included/excluded case reports. In the setting of disagreements, the individuals discussed the issue and, if needed, referred to a third author (AP) for a final decision.

### 2.2 Data Extraction and Quality Assessment

Data from included reports was extracted and compiled into a central database. Collected data included age, gender, name of ICI therapy, type of cancer, cardiac irAE, treatment, outcomes, and recommendations. Standardized Common Terminology Criteria for Adverse Events (CTCAE) v5.0 grading of irAEs was not feasible for many cases because most case reports lacked sufficient clinical detail. However, grading was possible for the small subset of reports in which rechallenge was discussed, as these typically included detailed symptom descriptions, biomarker data, and imaging findings. From the two databases, 1034 records were identified. The automation tool removed 584 for full text and abstract availability in English. A further 103 were removed because of duplication. An abstract review was conducted on 347 reports. Of this, 118 were removed because based on a review of the abstracts, the studies were determined not to be reporting a case report(s). These included randomized control trials (RCTs), reviews of RCTs, prospective and retrospective studies as well as reviews of retrospective and prospective studies. After excluding case reports that did not have abstracts available, full‐text options, or did not identify specific case report(s), 229 articles were identified for full article review. A full review of the 229 reports occurred; 32 cases reported combination therapy with an additional ICI or a chemotherapy agent. Additionally, a review of the full‐text availability of the reports yielded the further exclusion of 121 additional reports as they were determined not to be reporting cardiovascular irAEs associated with ICI therapy.

**FIGURE 1:**
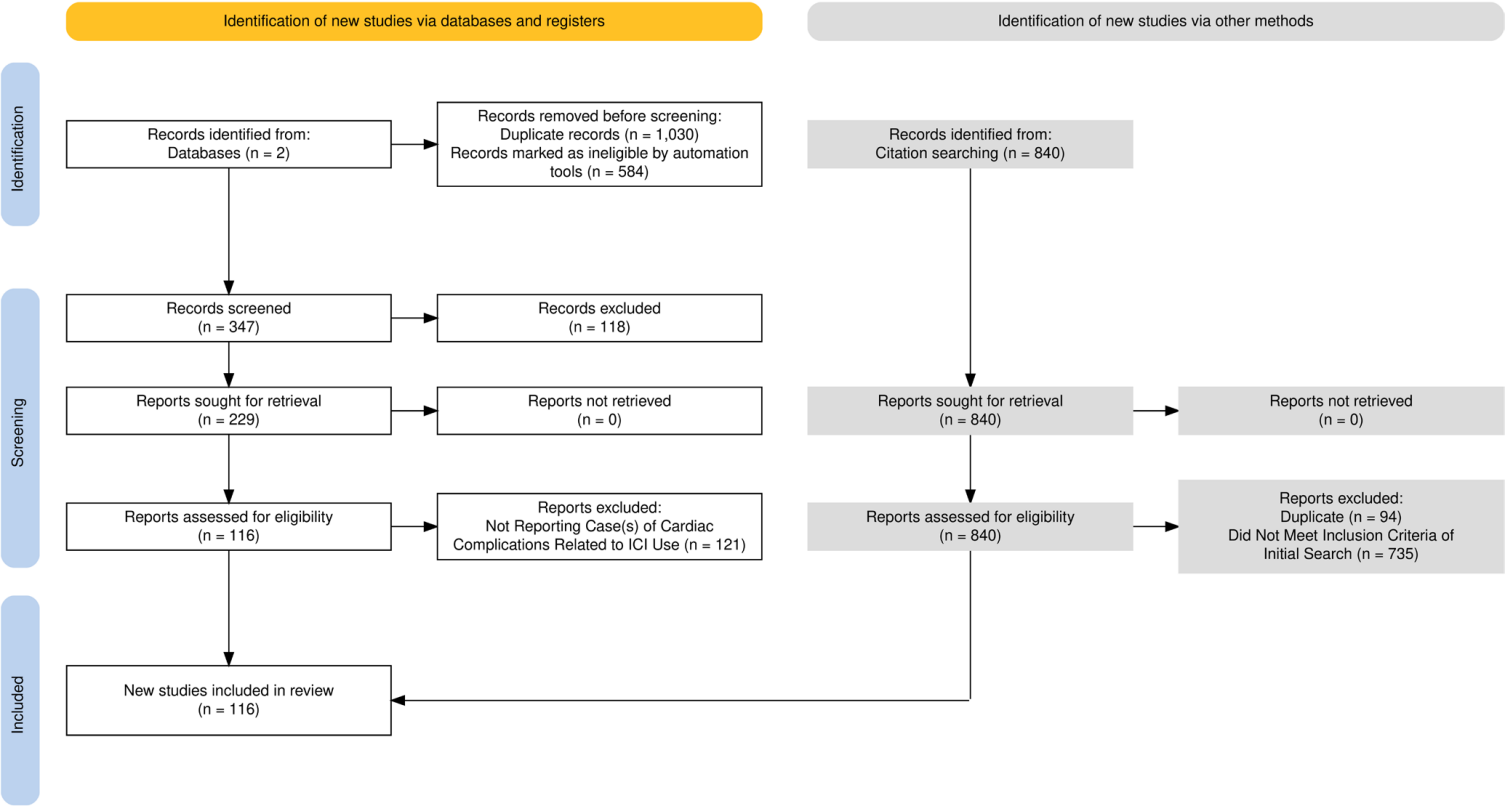
PRISMA flowchart showing study screening and selection process.

Subsequently, a manual search of the reference lists of the 110 case reports, including case reports describing combination therapy, was performed to identify any missed case reports. From this review, 15 non‐duplicate case studies were identified. They met the initial search criteria of full abstract and full text, specifying cardiovascular irAEs of ICI therapy with the three targeted therapies. In total, 84 case reports of monotherapy ICI utilization and 32 cases of combination ICI therapy were included in the study, amounting to 116 analyzed (Table 1). Case reports were assessed for quality using Pierson's five‐domain score, with a score greater than five out of a maximum possible score of 10 being considered consistent with a valid case report [13]. Figure 1 represents a PRISMA flowchart showing the study screening and selection process.

**TABLE 1:** Summarization of demographics, ICI drug name, cancer type, cardiovascular adverse event, treatments, and outcomes reported in individual case reports.

Study	Age	Gender	PMHx risk factors	Drug name(s)	Type of cancer	Complication	Treatment with corticosteroids	Corticosteroid type	Corticosteroid dosing (daily)	Discontinuation of ICI	Other treatment modalities utilized	Outcome
Abulnaja (2023) [98]	73	Female	None	Pembrolizumab	Lung Carcinoma	Myocarditis	Yes	Methylprednisolone	1000 mg	Yes	Beta Blocker	Alive
Ahmed et al. (2022) [108]	52	Male	Hypertension	Pembrolizumab + Paclitaxel	Lung Carcinoma	Heart Failure	Yes	Not Specified	Unspecified	Yes	GDMT, ACLS	Alive
Airò et al. (2022) [111]	77	Female	None	Pembrolizumab + Axitinib	Renal Cell Carcinoma	Heart Failure	Yes	Unspecified	Unspecified	Rechallenge	None	Alive
Alrayyashi et al. (2024) [94]	30	Female	None	Pembrolizumab	Rectal Carcinoma	Heart Failure	Yes	Prednisone	1 mg/kg	Yes	Unknown	Alive
Ansari‐Gilani et al. (2020) [88]	83	Male	None	Nivolumab	Renal Cell Carcinoma	Myocarditis and Heart Failure	Yes	Methylprednisolone	1 mg/kg	Yes	None	Death
Arangalage et al. (2021) [85]	61	Female	Hypertension	Pembrolizumab	Melanoma	Myocarditis and Heart Failure	Yes	Methylprednisolone	1000 mg	Rechallenge	ARB and Beta Blocker	Alive
Asai et al. (2019) [65]	62	Male	None	Nivolumab	Lung Carcinoma	Cardiac Tamponade	No	N/A	N/A	Rechallenge	Intraperitoneal Bleomycin and Pericardiocentesis	Alive
Atallah‐Yunes et al. (2019) [64]	66	Female	Atrial Fibrillation Hypertension	Pembrolizumab	Lung Carcinoma	Pericardial Effusion	Yes	Prednisone	60 mg	Yes	Pericardiocentesis	Alive
Baranseh et al. (2022) [123]	67	Female	Hypertension	Pembrolizumab	Lung Carcinoma	Heart Failure	No	N/A	N/A	Yes	GDMT	Alive
Barham et al. (2021) [73]	79	Female	None	Nivolumab + Ipilimumab	Melanoma	Myocarditis	Yes	Unspecified	Unspecified	Yes	None	Death
Behling et al. (2017) [49]	63	Male	Hypertension	Nivolumab	Melanoma	Heart Block	Yes	Prednisone	1.5 mg/kg	Yes	Antimicrobials	Death
Berkowitz et al. (2022) [154]	57	Male	None	Pembrolizumab	Lung Carcinoma	Cardiac Tamponade	Yes	Unspecified	Unspecified	Yes	Pericardial Window	Alive
Bharathidasan et al. (2023) [104]	54	Male	None	Nivolumab	Esophageal Carcinoma	Heart Failure	Yes	Methylprednisolone	1000 mg	Yes	Diuretics and Vasopressors and Inotropes and Antiarrhythmic Agents	Death
Bonhomme‐Faivre et al. (2023) [118]	65	Female	Hypertension	Nivolumab	Lymphoma	Heart Failure	Yes	Unspecified	Unspecified	Yes	GDMT	Alive
Brazel et al. (2023) [101]	60	Male	Ulcerative Colitis Hypertension	Nivolumab + Ipilimumab	Melanoma	Myocarditis and Heart Failure	Yes	Methylprednisolone	125 mg	Yes	GDMT and diuretics	Alive
Chatzantonis et al. (2020) [86]	30	Female	None	Pembrolizumab	Lung Carcinoma	Heart Failure	Yes	Methylprednisolone	1000 mg	Yes	Inotropes and Vasporessors and GDMT and Antiarrhythmic Agents and Temporary and Permanent Pacemaker and Mycophenolate	Alive
Chen et al. (2018) [90]	43	Male	None	Nivolumab	Thymic Carcinoma	Myocarditis	Yes	Methylprednisolone	1000 mg	Yes	None	Death
Dasanu et al. (2017) [79]	65	Female	None	Ipilimumab	Melanoma	Cardiac Tamponade	Yes	Methylprednisolone	240 mg	Yes	GDMT	Alive
de Almeida et al. (2018) [56]	69	Male	None	Nivolumab	Lung Carcinoma	Cardiac Tamponade	Yes	Prednisone	1 mg/kg	Yes	Pericardiocentesis and Pericardial Window	Alive
Delgado‐Lazo et al. (2022) [117]	43	Male	None	Pembrolizumab	Colon Carcinoma	Myocarditis	Yes	Methylprednisolone	125 mg	Yes	GDMT and Antimicrobials	Alive
Delombaerde et al. (2022) [116]	69	Male	None	Nivolumab + Ipilimumab	Cholangiocarcinoma	Myocarditis	Yes	Prednisolone	1.5 mg/kg	Yes	None	Alive
Di Marco et al. (2024) [93]	57	Female	None	Pembrolizumab + Axitinib	Renal Cell Carcinoma	Heart Failure	Yes	Unspecified	Unspecified	Yes	Dobutamine, Levosimendan	Death
Duarte et al. (2022) [112]	67	Female	Hypertension	Pembrolizumab	Bladder Carcinoma	Myocarditis and Heart Failure	Yes	Prednisolone	2 mg/kg	Yes	GDMT	Death
Ederhy et al. (2021) [135]	60	Female	None	Nivolumab + Ipilimumab	Lung Carcinoma	Myocarditis	Yes	Unspecified	Unspecified	Yes	Plasmapheresis	Alive
Fazal et al. (2020) [72]	82	Male	None	Nivolumab	Melanoma	Myocarditis	Yes	Methylprednisolone	1000 mg	Yes	Pyridostigmine	Death
Fazel and Jedlowski (2019) [75]	78	Female	Hypertension	Nivolumab + Ipilimumab	Melanoma	MMM Overlap Syndrome	Yes	Methylprednisolone	1 mg/kg	Yes	IVIG	Death
Fernández Madrigal et al. (2022) [155]	62	Female	None	Pembrolizumab + Cisplatin + Pemetrexed	Lung Carcinoma	Cardiac Tamponade	Yes	Unspecified	1 mg/kg	Yes	Pericardiocentesis with pericardial drain	Alive
Fratti et al. (2023) [99]	72	Male	CKD	Pembrolizumab + Axitinib	Renal Cell Carcinoma	Myocarditis and Heart Failure	Yes	Methylprednisolone	1000 mg	Yes	GDMT	Alive
Frigeri et al. (2017) [53]	76	Female	None	Nivolumab	Lung Carcinoma	Myocarditis	Yes	Methylprednisolone	5 mg/kg	Yes	Unknown	Alive
Fukasawa et al. (2017) [78]	69	Female	None	Nivolumab	Lung Carcinoma	Myocarditis and Myasthenia Gravis	Yes	Methylprednisolone	1 mg/kg	Yes	Temporary Pacing	Alive
Gallegos et al. (2019) [69]	47	Female	Carotid artery dissection	Nivolumab	Melanoma	Myocarditis	Yes	Methylprednisolone	1000 mg	Yes	Unknown	Death
Ganatra and Neilan (2018) [76]	41	Female	Hashimoto Thyroiditis	Nivolumab + Ipilimumab	Melanoma	Myocarditis	Yes	Methylprednisolone	1000 mg	Yes	Neurohormonal Antagonists	Alive
Gibson et al. (2016) [46]	68	Female	Wolff‐Parkinson‐White	Nivolumab	Lung Carcinoma	Myocarditis	Yes	Methylprednisolone	80 mg	Rechallenge	Antiarrhythmic Agents	Death
Giovannini et al. (2023) [102]	65	Male	None	Pembrolizumab	Melanoma	Myocarditis and Heart Block	Yes	Methylprednisolone	60 mg	Yes	Pyridostigmine	Death
Harada et al. (2020) [142]	63	Female	None	Pembrolizumab	Lung Carcinoma	Cardiac Tamponade	No	N/A	N/A	Rechallenge	Pericardiocentesis	Death
Hardy et al., (2020) [146]	81	Male	Lung carcinoma, removed	Nivolumab + Ipilimumab	Renal Cell Carcinoma	Myocarditis	Yes	Methylprednisolone	1 mg/kg	Yes	None	Death
Hellman et al. (2019) [60]	83	Male	None	Pembrolizumab + Epacadostat	Urothelial Cell Carcinoma	Myocarditis	Yes	Prednisone	1 mg/kg	Yes	None	Death
Hsu et al. (2018) [61]	42	Male	None	Pembrolizumab	Hepatocellular Carcinoma	Sick Sinus Syndrome	Yes	Cortisone	12.5 mg	Yes	None	Alive
Huertas et al. (2019) [89]	80	Male	Hypertension	Nivolumab	Renal Cell Carcinoma	Myocarditis	Yes	Methylprednisolone	2 mg/kg	Yes	None	Death
Ida et al. (2022) [120]	81	Female	Hypertension	Nivolumab + Ipilimumab	Melanoma	Myocarditis	Yes	Prednisolone	1.5 mg/kg	Yes	IVIG	Alive
Inayat et al. (2018) [54]	74	Male	Hypertension CVA	Pembrolizumab	Lung Carcinoma	Heart Block	Yes	Prednisone	1 mg/kg	Yes	DAPT and Beta Blocker	Death
Iniesta et al. (2020) [136]	69	Male	Urothelial Cancer in remission	Pembrolizumab	Lung Carcinoma	Myocarditis	Yes	Methylprednisolone	1 mg/kg	Yes	Diuretics and Vasopressors	Alive
Jain et al. (2018) [59]	67	Male	None	Nivolumab + Ipilimumab	Melanoma	Myocarditis	Yes	Prednisone	1 mg/kg	Yes	PPM	Death
Jang et al. (2022) [114]	48	Female	Myasthenia gravis Thymectomy	Pembrolizumab	Thymic Carcinoma	Myocarditis and Myositis	Yes	Methylprednisolone	2 mg/kg	Yes	Plasmapheresis and IVIG	Alive
Jespersen et al. (2021) [124]	57	Male	None	Nivolumab + Ipilimumab	Renal Cell Carcinoma	Myocarditis and Heart Block	Yes	Methylprednisolone	2 mg/kg	Yes	Abatacept and Mycophenolate mofetil	Alive
Johnson et al. (2016) [48]	65	Male	None	Nivolumab + Ipilimumab	Melanoma	Myocarditis	Yes	Methylprednisolone	2 mg/kg	Yes	None	Death
Johnson et al. (2016) [48]	63	Female	None	Nivolumab + Ipilimumab	Melanoma	Myocarditis	Yes	Methylprednisolone	1000 mg	Yes	None	Death
Katsume et al. (2018) [58]	73	Male	None	Pembrolizumab	Lung Carcinoma	Heart Block	Yes	Methylprednisolone	1000 mg	Yes	Permanent Pacemaker	Alive
Khachatyran et al. (2023) [100]	44	Female	HFrEF Postural Tachycardia Syndrome	Pembrolizumab	Lymphoma	Cardiac Tamponade	Yes	Methylprednisolone	1 mg/kg	Yes	Pericardiocentesis	Alive
Khan et al. (2019) [149]	62	Male	None	Pembrolizumab	Lung Carcinoma	Cardiac Tamponade	Yes	Methylprednisolone	1 mg/kg	Yes	Pericardial Window	Alive
Khan et al. (2020) [147]	67	Female	Hypertension	Pembrolizumab	Lung Carcinoma	Myocarditis and Heart Block	No	N/A	N/A	Yes	Temporary Pacing and Permanent Pacemaker	Alive
Khoury et al. (2019) [152]	68	Male	Hypertension Prostate Cancer in remission	Nivolumab + Ipilimumab	Melanoma	Myocarditis	Yes	Prednisone	2 mg/kg	Yes	None	Alive
Komatsu et al. (2021) [129]	66	Male	None	Nivolumab	Gastric Carcinoma	Myocarditis	Yes	Methylprednisolone	1 mg/kg	Yes	Plasmapheresis and IVIG	Death
Kumamoto et al. (2022) [125]	54	Female	None	Nivolumab	Hypopharyngeal cancer	Vasospastic Angina	No	N/A	N/A	Rechallenge	Antiarrhythmic Agents	Alive
Kushnir and Wolf (2017) [47]	67	Male	None	Nivolumab	Lung Carcinoma	Cardiac Tamponade	Yes	Unspecified	Unspecified	Yes	Pericardiocentesis	Alive
Kwan et al. (2019) [63]	71	Female	Hypertension	Pembrolizumab	Giant Cell Bone Tumor	Acute Coronary Syndrome	No	N/A	N/A	Yes	Cardiac Catheterization	Alive
Lam et al. (2022) [92]	71	Male	Hypertension	Pembrolizumab	Lymphoma	Myocarditis	Yes	Methylprednisolone	1 mg/kg	Yes	Mycophenolate	Death
Läubli et al. (2015) [81]	73	Female	None	Pembrolizumab	Melanoma	Heart Failure	Yes	Prednisone	2 mg/kg	Yes	GDMT	Alive
Leaver et al. (2020) [140]	55	Male	Hypertension	Nivolumab + Ipilimumab	Melanoma	Myocarditis and Myasthenia Gravis	Yes	Prednisone	1 mg/kg	Yes	IVIG	Alive
Lee et al. (2020) [145]	31	Female	None	Pembrolizumab	Sarcoma	Myocarditis	Yes	Prednisone	80 mg	Rechallenge	None	Alive
Lewis et al. (2024) [95]	57	Female	None	Pembrolizumab	Lung Carcinoma	Myocarditis	Yes	Prednisone	1 mg/kg	Yes	GDMT	Alive
Li et al. (2021) [126]	62	Male	Hypertension Coronary Artery Disease	Pembrolizumab	Lung Carcinoma	Myocarditis	Yes	Methylprednisolone	80 mg	Yes	Continuous Renal Replacement Therapy	Alive
Loong Tan et al. (2020) [138]	62	Male	Cirrhosis	Nivolumab	Hepatocellular Carcinoma	Myocarditis and Heart Failure	Yes	Methylprednisolone	2 mg/kg	Yes	None	Alive
Masson et al. (2020) [87]	62	Male	Coronary Artery Disease	Nivolumab	Melanoma	Acute Coronary Syndrome	No	N/A	N/A	Yes	Cardiac Catheterization and CABG	Alive
Matson et al. (2018) [50]	55	Male	None	Nivolumab	Lung Carcinoma	Myocarditis	Unknown	N/A	N/A	Yes	Unknown	Death
Matsui et al. (2020) [137]	69	Male	None	Pembrolizumab	Bladder Carcinoma	Myocarditis and Myositis	Yes	Methylprednisolone	1 mg/kg	Yes	Plasmapheresis and ECMO and IABP	Death
Matsuo et al. (2019) [71]	62	Male	None	Nivolumab	Lung Carcinoma	Myocarditis	Yes	Prednisolone	1 mg/kg	Yes	None	Alive
Miyauchi et al. (2021) [127]	71	Male	Hypertension	Nivolumab + Ipilimumab	Renal Cell Carcinoma	Myocarditis	Yes	Prednisolone	1 mg/kg	Yes	None	Alive
Mohammad et al. (2023) [82]	32	Female	None	Pembrolizumab	Breast Carcinoma	Myocarditis	Yes	Methylprednisolone	500 mg	Yes	Abatacept	Alive
Monge et al. (2018) [62]	79	Male	Atrial Fibrillation	Nivolumab + PROSTVAC	Prostate Carcinoma	Myocarditis	Yes	Methylprednisolone	1 mg/kg	Yes	None	Alive
Moriyama et al. (2021) [133]	58	Male	None	Nivolumab	Lung Carcinoma	Pericarditis	Yes	Prednisolone	1 mg/kg	Yes	Infliximab	Alive
Naganuma et al. (2022) [121]	74	Male	Bladder Cancer	Nivolumab	Gastric Carcinoma	Myocarditis	Yes	Prednisolone	1 mg/kg	Yes	IVIG and Impella	Death
Nierstedt et al. (2020) [144]	77	Male	Hypertension	Pembrolizumab	Lung Carcinoma	Myocarditis	Yes	Unspecified	Unspecified	Yes	Antiarrhythmic Agents	Death
Nishikawa et al. (2022) [115]	78	Male	None	Pembrolizumab + Bevacizumab	Lung Carcinoma	Myocarditis	Yes	Prednisolone	1 mg/kg	Yes	None	Alive
Nishimura et al. (2023) [113]	59	Male	None	Nivolumab + Ipilimumab	Lung Carcinoma	Myocarditis	Yes	Methylprednisolone	1000 mg	Yes	None	Death
Norwood et al. (2017) [52]	49	Female	None	Nivolumab + Ipilimumab	Melanoma	Myocarditis	Yes	Unspecified	Unspecified	Yes	None	Alive
Ono et al. (2022) [84]	77	Female	None	Nivolumab	Lung Carcinoma	MMM Overlap Syndrome	Yes	Prednisone	80 mg	Yes	Temporary Pacing	Death
Oristrell et al. (2018) [68]	55	Female	None	Pembrolizumab	Breast Carcinoma	Cardiac Tamponade	Yes	Unspecified	2 mg/kg	Rechallenge	Pericardiocentesis	Alive
Pollock and Castillo (2023) [103]	69	Male	None	Pembrolizumab	Lung Carcinoma	Pericardial Effusion	No	N/A	N/A	Rechallenge	Pericardial Window	Alive
Portolés Hernández et al. (2021) [128]	48	Female	None	Pembrolizumab	Thymic Carcinoma	Myocarditis and Heart Block	Yes	Methylprednisolone	2 mg/kg	Yes	IV Antithymocyte Globulin Antiarrhythmic Agents and Permanent Pacemaker	Death
Prevel et al. (2020) [148]	80	Male	Hypertension	Nivolumab	Lung Carcinoma	Myocarditis and Heart Block	Yes	Methylprednisolone	1000 mg	Yes	Temporary Pacing	Death
Rhee et al. (2022) [83]	73	Male	Right Bundle Branch Block	Nivolumab	Melanoma	MMM Overlap Syndrome	Yes	Prednisone	60 mg	Yes	IVIG	Alive
Roth et al. (2016) [45]	60	Male	Hypertension	Ipilimumab	Melanoma	Heart Failure	No	N/A	N/A	Yes	ACEi and Beta Blocker	Alive
Saad et al. (2024) [97]	82	Female	Hypertension	Pembrolizumab	Bladder Carcinoma	Myocarditis and Heart Block	Yes	Methylprednisolone	1 mg/kg	Yes	Pacemaker	Alive
Saade et al. (2019) [77]	58	Female	None	Nivolumab	Lung Carcinoma	Cardiac Tamponade	Yes	Prednisone	1 mg/kg	Yes	Pericardiocentesis	Death
Saade et al. (2019) [77]	65	Male	None	Nivolumab	Lung Carcinoma	Cardiac Tamponade	Yes	Unspecified	Unspecified	Rechallenge	Pericardiocentesis	Alive
Saade et al. (2019) [77]	55	Female	None	Nivolumab	Lung Carcinoma	Cardiac Tamponade	Yes	Unspecified	Unspecified	Yes	Pericardiocentesis	Death
Saibil et al. (2019) [74]	67	Male	Hypertension	Nivolumab + Ipilimumab	Melanoma	Myocarditis and Myositis	Yes	Methylprednisolone	1000 mg	Yes	Infliximab and Tocilizumab	Death
Sakai et al. (2019) [70]	74	Male	None	Nivolumab	Lung Carcinoma	Heart Failure	No	N/A	N/A	Yes	ECMO	Death
Samara et al. (2019) [55]	77	Male	Hypertension CKD	Ipilimumab	Melanoma	Myocarditis	Yes	Methylprednisolone	120 mg	Yes	None	Death
Samejima et al. (2020) [32]	79	Male	None	Nivolumab	Lung Carcinoma	Heart Failure	No	N/A	N/A	Yes	Inotropes and GDMT	Alive
Schiopu et al. (2021) [132]	75	Male	None	Pembrolizumab	Lung Carcinoma	Myocarditis	Yes	Prednisone	1 mg/kg	Yes	Cardioversion and Plasmapheresis	Alive
Semper et al. (2016) [91]	75	Male	None	Nivolumab	Lung Carcinoma	Myocarditis	Yes	Prednisolone	1 mg/kg	Yes	GDMT	Alive
Shalata et al. (2024) [96]	74	Female	None	Nivolumab + Ipilimumab	Lung Carcinoma	Myopericarditis	Yes	Prednisone	2 mg/kg	Yes	Colchicine	Alive
Sharma et al. (2019) [67]	76	Female	Psoriatic arthritis	Nivolumab	Angioimmunoblastic T‐cell lymphoma (AITL)	Heart Failure	Yes	Methylprednisolone	1 mg/kg	Yes	Vasopressors and Impella	Death
Shindo et al. (2022) [122]	79	Male	None	Nivolumab	Gastric Carcinoma	Myocarditis	Yes	Prednisolone	30 mg	Yes	None	Death
Su et al. (2022) [119]	80	Female	None	Pembrolizumab	Head and Neck Squamous Cell Carcinoma	Myocarditis and Heart Block	Yes	Methylprednisolone	1 mg/kg	Yes	Permanent Pacemaker	Alive
Szuchan et al. (2020) [139]	70	Female	None	Pembrolizumab	Thymic Carcinoma	Myocarditis and Myasthenia Gravis	Yes	Prednisone	1 mg/kg	Yes	Plasmapheresis and Pyridostigmine	Alive
Tachihara et al. (2019) [66]	70	Male	None	Pembrolizumab	Lung Carcinoma	Cardiac Tamponade	No	N/A	N/A	Rechallenge	Pericardiocentesis	Alive
Tajmir‐Riahi et al. (2018) [51]	72	Male	None	Nivolumab + Ipilimumab	Melanoma	Heart Failure	Yes	Prednisolone	1 mg/kg	Yes	None	Death
Takai et al. (2020) [141]	77	Male	None	Pembrolizumab	Bladder Carcinoma	Myocarditis and Myasthenia Gravis	Yes	Prednisone	80 mg	Yes	IVIG	Death
Tan et al. (2019) [151]	74	Male	None	Nivolumab	Lung Carcinoma	Myocarditis and Heart Block	Yes	Prednisolone	1 mg/kg	Yes	ACEi and Beta Blocker and Permanent Pacemaker	Alive
Tanriverdi et al. (2020) [143]	70	Male	None	Pembrolizumab + Axitinib	Renal Cell Carcinoma	Heart Failure	No	N/A	N/A	Rechallenge	Diuretics, Acetylcysteine, LMWH	Alive
Tsuruda et al. (2019) [150]	47	Male	None	Nivolumab	Ethmoid Sinus Carcinoma	Myocarditis	Yes	Prednisolone	1000 mg	Yes	IVIG	Death
Uchida et al. (2022) [109]	69	Male	Coronary Artery Disease	Pembrolizumab	Lung Carcinoma	Cardiac Tamponade	No	N/A	N/A	Rechallenge	Pericardiocentesis	Alive
Uczkowski et al. (2023) [106]	54	Male	None	Pembrolizumab	Lung Carcinoma	Cardiac Tamponade	No	N/A	N/A	Yes	Pericardial Window	Alive
Vartanov et al. (2021) [130]	78	Male	Hypertension Coronary Artery Disease	Nivolumab + Ipilimumab	Lung Carcinoma	Heart Block	No	N/A	N/A	Yes	Offered PPM—declined	Death
Verhaert et al. (2023) [107]	50	Female	None	Pembrolizumab	Melanoma	Pericarditis	Yes	Methylprednisolone	1 mg/kg	Yes	Azathioprine	Alive
Xie et al. (2021) [134]	67	Male	None	Pembrolizumab	Lung Carcinoma	MMM Overlap Syndrome	Yes	Methylprednisolone	1 mg/kg	Yes	Antimicrobials and Temporary Pacing	Alive
Yamaguchi et al. (2018) [57]	60	Male	None	Nivolumab	Melanoma	Myocarditis	Yes	Prednisolone	1000 mg	Yes	ECMO and IVIG	Alive
Yanase et al. (2020) [153]	59	Male	None	Nivolumab + Ipilimumab	Renal Cell Carcinoma	Myocarditis and Myasthenia Gravis	Yes	Methylprednisolone	1000 mg	Yes	IVIG and Plasma Exchange	Alive
Yogasundaram et al. (2021) [131]	69	Male	Hypertension TIA	Pembrolizumab	Prostate Carcinoma	Myocarditis and Heart Failure	Yes	Methylprednisolone	1000 mg	Yes	Mycophenolate and Plasmapheresis	Death
Yun et al. (2015) [80]	59	Male	None	Ipilimumab	Melanoma	Myocarditis	Yes	Methylprednisolone	125 mg	Yes	Indomethacin and Pericardiocentesis	Alive
Zhou et al. (2022) [156]	67	Male	Hypertension	Pembrolizumab	Lung Carcinoma	Heart Failure	Yes	Methylprednisolone	160 mg	Yes	GDMT	Alive
Zhang et al. (2023) [105]	55	Male	Ankylosing Spondylitis	Pembrolizumab + Lenvatinib	Cholangiocarcinoma	Pulmonary Arterial Hypertension	Yes	Methylprednisolone	20 mg	Rechallenge	Mycophenolate mofetil	Alive
Zomborska et al. (2022) [110]	59	Female	None	Nivolumab	Thymic Carcinoma	Myocarditis and Heart Failure	Yes	Methylprednisolone	1000 mg	Yes	Mycophenolate	Death

### 2.3 Data Synthesis and Analysis

We summarized the available literature, reporting baseline participant characteristics, the implicated ICI, therapeutic interventions, and clinical outcomes. All statistical analyses were performed using Tableau version 2024.1 (Seattle, Washington).

## 3 Results

### 3.1 Demographic Summary

**FIGURE 2:**
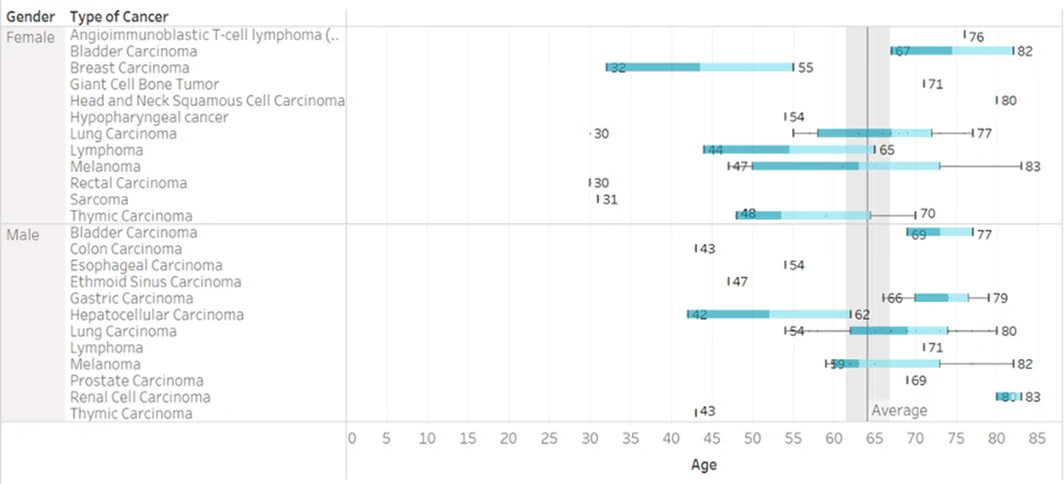
Box plot demographics of monotherapy case reports. This figure depicts the breakdown between males and females with reported cancer types and the age range and average of the reported types within each cancer type based on gender. The figure also depicts the average age (dark grey line) of the patients as well as the standard deviation of the age (grey shadowed areas). The intersection of the dark blue and the light blue boxes depict the median age of the specified cancer group.

**FIGURE 3:**
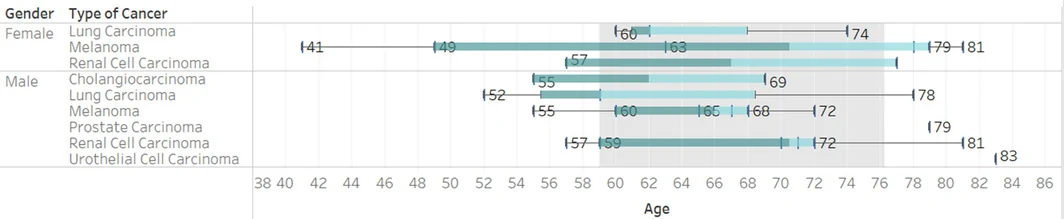
Box plots demographics of combination therapy case reports. This figure depicts the breakdown between males and females with reported cancer types and the age range and average of the reported types within each cancer type based on gender. The figure also depicts the upper and lower quartiles of the age range (grey shadowed areas). The intersection of the dark blue and the light blue boxes depict the median age of the specified cancer group.

We present the analysis of 116 case reports from 115 studies, including 84 cases of ICI monotherapy and 32 cases of combination therapy. Figures 2 and 3 summarize the demographics of the reported cases, including the biological sex, age, and type of cancer. The average age of reported patients was 64 years in monotherapy utilization (62 years for females, 65 years for males) and 67 years (66 years for females, 67 years for males) for combination therapy utilization. Further details pertaining to study characteristics, cancer type, ICI administered, and demographics are illustrated in Figures 2 and 3.

### 3.2 Clinical Spectrum of Observed Cardiovascular Immune‐Related Adverse Events (irAEs)

Myocarditis was the most frequently reported and severe complication. Among monotherapy cases, 48 instances were identified, with nivolumab and pembrolizumab being the most frequently implicated ICIs. Myocarditis was isolated in 27 cases, with corticosteroids being the primary treatment. Mortality in these cases was notably high at 48.1% (13/27), particularly with nivolumab where 71.4% (10/14) of patients experienced death. Additionally, there were 21 reported instances of myocarditis occurring concomitantly with another associated syndrome, such as heart block (7 cases), heart failure (6 cases), myasthenia gravis (6 cases, 3 of which are myocarditis, myositis, and myasthenia gravis (MMM) Overlap Syndrome), and myositis (4 cases, 2 of which are MMM Overlap Syndrome), further complicating the management and leading to increased mortality, particularly in severe cases. In combination therapy, 25 cases were documented, mostly involving nivolumab/ipilimumab. Treatment generally consisted of corticosteroids, with mortality occurring in seven cases, predominantly within the nivolumab/ipilimumab group.

Heart failure was reported in 11 monotherapy cases, primarily involving pembrolizumab and nivolumab. These patients were treated with corticosteroids and guideline‐directed medical therapy (GDMT). Three of these cases resulted in mortality. Five cases were reported in combination therapy, including nivolumab/ipilimumab and pembrolizumab/axitinib combinations. Mortality was observed in two cases, indicating a continued risk of death despite management with corticosteroids and GDMT.

Heart blocks also emerged as a notable issue, most commonly after monotherapy, including pembrolizumab or nivolumab, and often seen as a sequela of myocarditis. There were 10 cases of heart block associated with monotherapy; seven of these cases were seen in conjunction with myocarditis. These cases were treated with corticosteroids and, in some instances, permanent pacemakers or other adjunctive therapies, including beta blockade. Six patients required pacing for pacemaker therapy, with five of these patients ultimately receiving permanent pacemakers. Three of these patients died, underscoring the severity of the condition. The only combination therapy case of isolated heart block, involving nivolumab/ipilimumab, was offered treatment with a permanent pacemaker. However, the patient declined and subsequently died. There was another case of heart block associated with myocarditis after treatment with nivolumab/ipilimumab, which resolved with treatment using corticosteroids and abatacept.

Cardiac tamponade was another complication observed in 15 monotherapy cases, particularly with pembrolizumab and nivolumab. Treatments included corticosteroids, pericardiocentesis, and pericardial window, with mortality occurring in three cases. Fortunately, 80% of the patients treated survived. There was also one case in combination therapy, which was successfully treated with corticosteroids and a pericardiocentesis with pericardial drain.

Finally, rare complications such as Takotsubo cardiomyopathy, acute coronary syndrome, and pulmonary arterial hypertension were observed. These cases had favorable outcomes, with no mortality in cases of Takotsubo cardiomyopathy or acute coronary syndrome and one successful rechallenge in a case of pulmonary arterial hypertension. Other less common syndromes were also noted in this analysis, including MMM overlap syndrome, with a few cases requiring additional immunosuppressive therapies such as infliximab and plasmapheresis. While the majority of these patients survived, there were a few cases of mortality.

**FIGURE 4:**
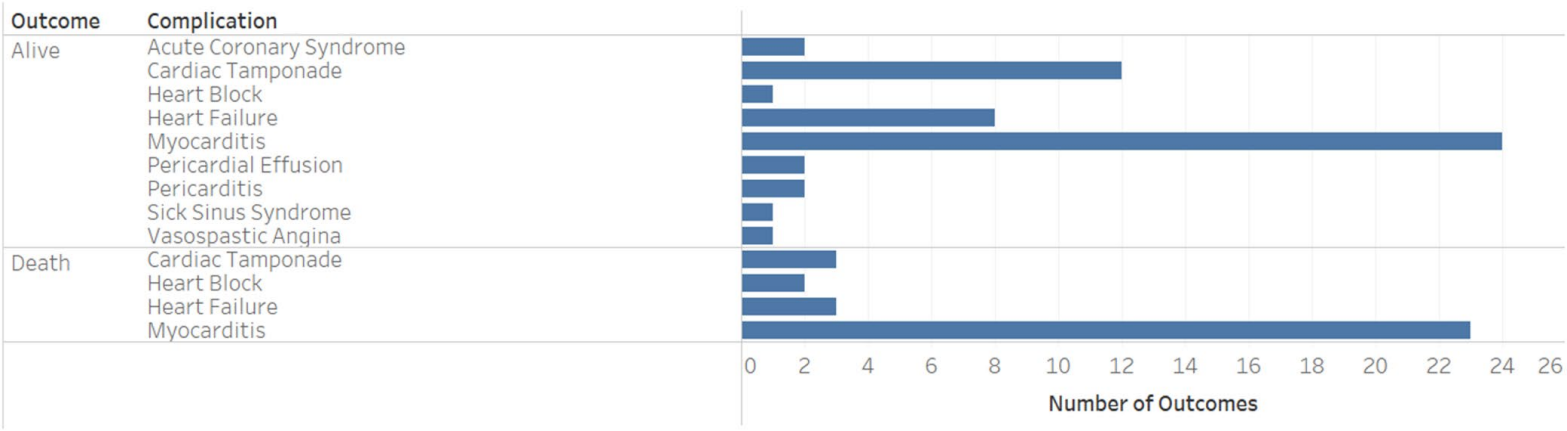
Outcomes of reported cardiovascular irAEs in monotherapy cases. This figure shows the outcome, alive or dead, of each cardiovascular irAE type reported.

**FIGURE 5:**
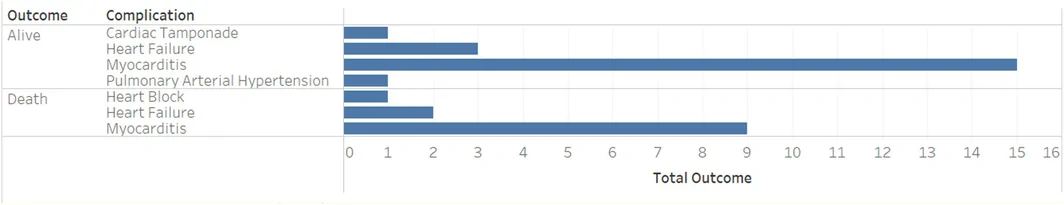
Outcomes of reported cardiovascular irAEs in combination therapy cases. This figure shows the outcome, alive or dead, of each cardiovascular irAE type reported.

**FIGURE 6:**
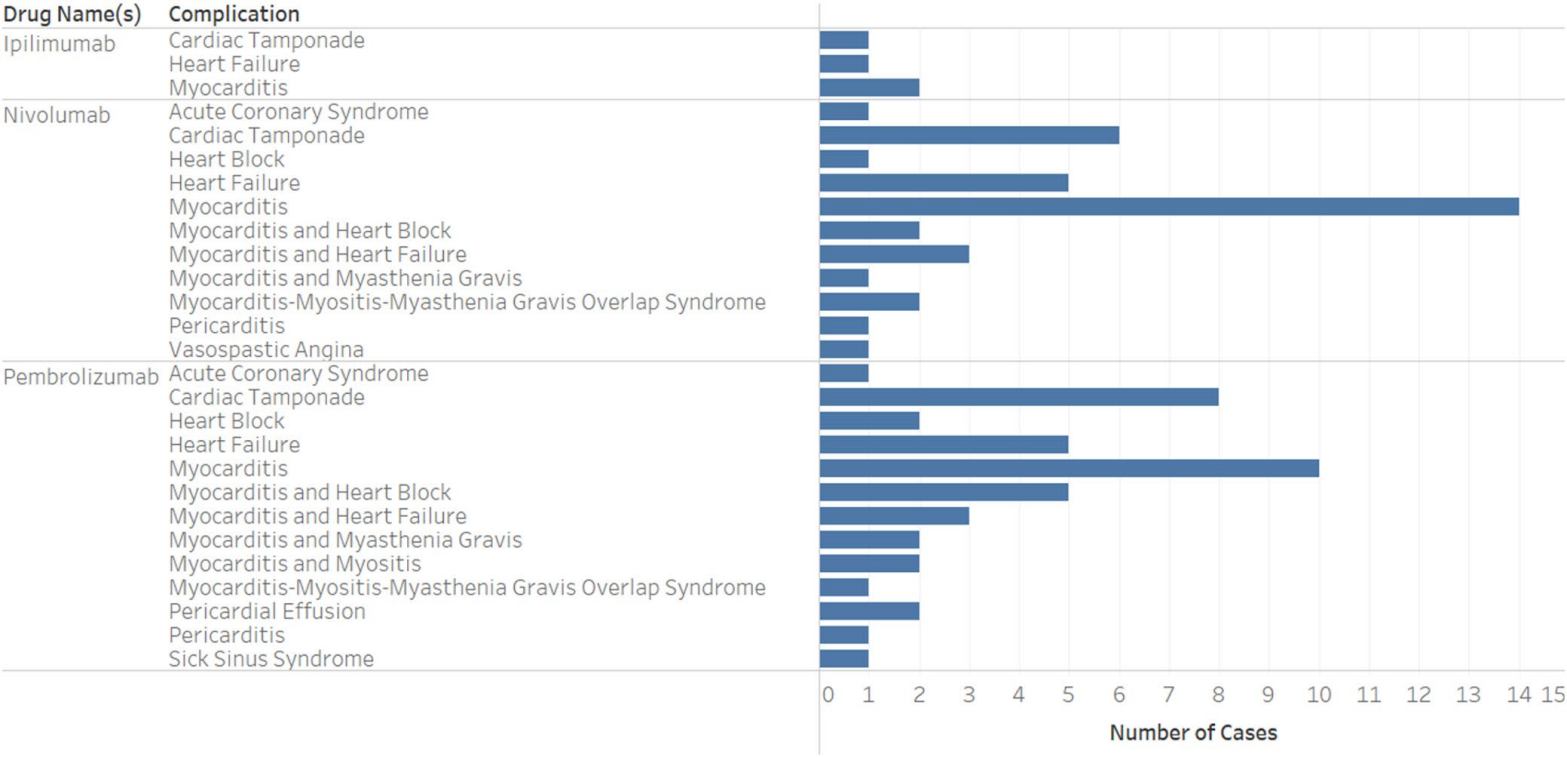
Immune checkpoint inhibitor reported cardiovascular irAEs in monotherapy cases. This figure depicts the reported cardiovascular irAEs of each immune checkpoint inhibitor therapy type.

**FIGURE 7:**
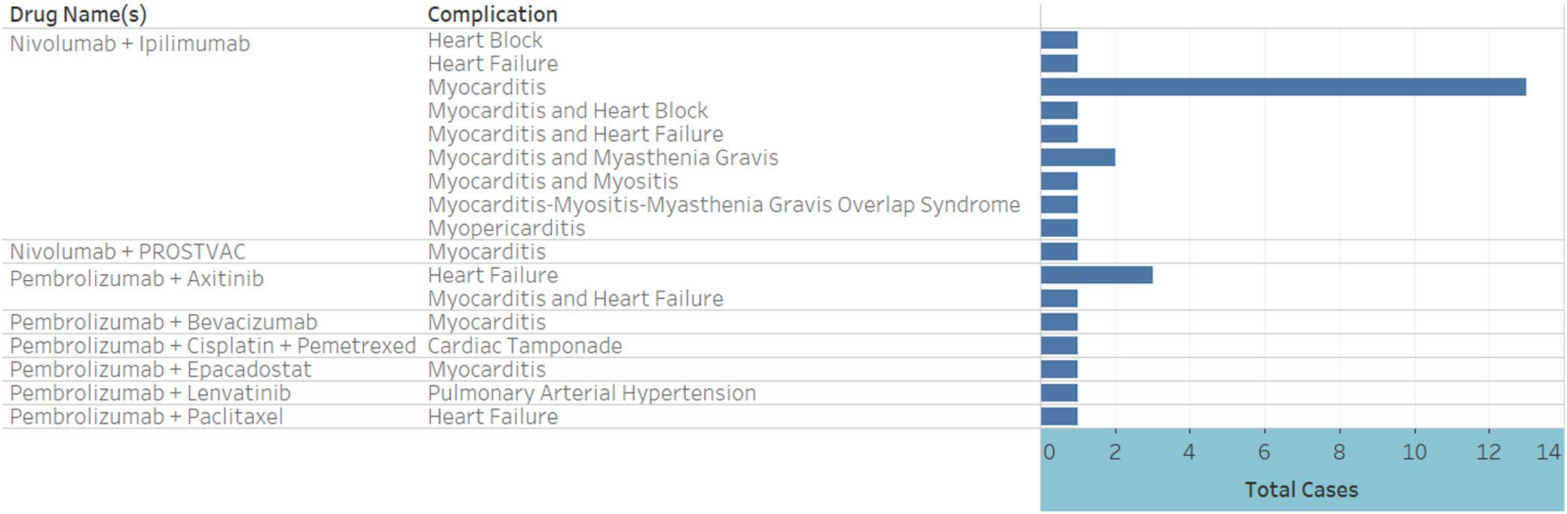
Immune checkpoint inhibitor reported cardiovascular irAEs in combination therapy cases. This figure depicts the reported cardiovascular irAEs of each immune checkpoint inhibitor therapy type.

Figures 4 and 5 show the outcome (alive or dead) of reported cardiovascular irAE in the monotherapy and combination therapy cases, respectively. Figures 6 and 7 further break down the outcome by type of ICI utilized in monotherapy and combination therapy with outcome, respectively.

## 4 Results of Analysis of Outcomes

### 4.1 Treatment Insights

#### 4.1.1 Corticosteroid Use

Of the 116 cases examined, 99 patients (85.3%) were treated with corticosteroids, 16 (13.7%) were not, and one case was unknown. Various dosing patterns were utilized: a combination of high‐dose (> 1 mg/kg), pulse‐dose steroids (69%), or moderate‐to‐low‐dose steroids (20%). Eleven cases had unspecified corticosteroid dosing. Dosing for high‐dose steroids varied, with one patient treated with 5 mg/kg, 11 treated with 2 mg/kg, three with 1.5 mg/kg, and 35 with 1 mg/kg, the standard dosing. Additionally, 19 patients received 1‐g pulse‐dose steroids, primarily with methylprednisolone.

Among the 39 patients treated with high‐dose or pulse steroids (intravenous methylprednisolone 1 g/day), 37 remained alive: 21 were treated with 1 mg/kg/day, 2 with 1.5 mg/kg/day, 1 with 5 mg/kg/day, 7 with 2 mg/kg/day, and 8 with 1 g/day pulse dose. Fatalities included 14 patients with 1 mg/kg/day standard dosing, one with 1.5 mg/kg/day, 4 with 2 mg/kg/day, and 11 who received 1 g/day pulse dose. Patients managed with high‐dose steroids experienced a favorable outcome in 53.6% of cases.

Evaluating patients treated with steroids above the recommended 1 mg/kg/day, 15 patients (21.7%) received > 1 mg/kg/day, aside from pulse‐dose steroids. Of these cases, 10 (66.6%) had favorable outcomes. Regarding low‐to‐moderate dose steroids, 18 cases involved doses below the standard 1 mg/kg or pulse‐dose steroid recommendation, ranging from 12.5 to 500 mg daily. Of these, 14 (77.7%) experienced favorable outcomes.

Sixteen patients did not receive corticosteroids as part of their treatment regimen. Of these cases, 81.3% experienced favorable outcomes (survival); however, several patients received cardiac treatments including GDMT for heart failure, antiarrhythmic agents for arrhythmias, as well as procedural interventions including cardiac catheterization, temporary pacing, permanent pacemakers, pericardiocentesis, and pericardial window for emergent management, with some patients undergoing multiple procedures.

**FIGURE 8:**
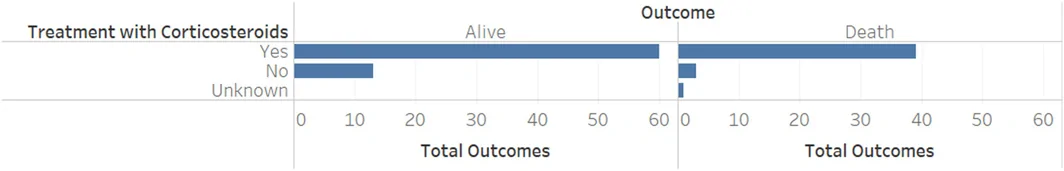
Corticosteroid utilization in cardiovascular irAEs and reported survival outcomes. This figure depicts the reported corticosteroid utilization in cardiovascular irAEs and the survival outcome based on utilization.

**FIGURE 9:**
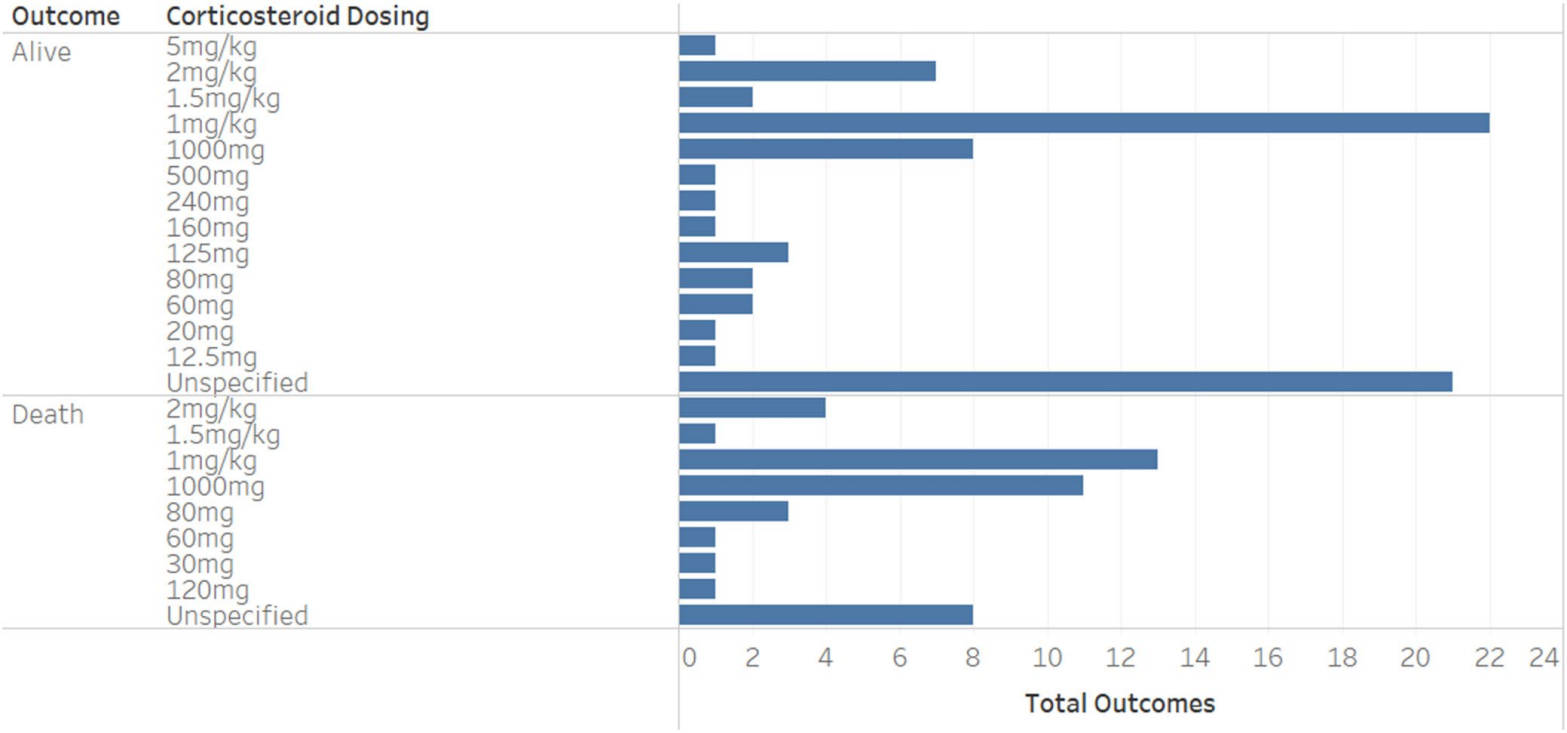
Corticosteroid dosing utilization in cardiovascular irAEs and reported survival outcomes. This figure depicts the reported corticosteroid utilization and dosing in cardiovascular irAEs and the survival outcomes.

Among the analyzed reports, survival appeared to be higher for patients treated with high‐dose steroids than with low‐moderate doses (94.8% vs. 66.6%) suggesting that the former may be preferable. Figure 8 depicts the relationship between steroid use and outcome, while Figure 9 depicts steroid use and dosing with the outcome of the analyzed cases.

#### 4.1.2 Adjunctive Therapies

##### 4.1.2.1 Intravenous Immunoglobulin (IVIG)

IVIG was used in 11 cases, with reported survival rates of 54.5%. In 81.8% of these cases, IVIG was administered alongside high‐dose/pulse steroids. The combination of IVIG and high‐dose steroids was beneficial, as a higher proportion of survivors were treated with both therapies. Of the patients treated with combined high‐dose/pulse steroids alongside IVIG, 55.5% survived. This is compared to the patients treated with low/moderate dose steroids along with IVIG, where 50% of the patients survived; however, it should be noted that only two patients were treated with lower doses of steroids. This suggests that the combination of IVIG and high‐dose steroids may improve patient outcomes in cases requiring adjunctive therapy.

##### 4.1.2.2 Plasmapheresis

Plasmapheresis was utilized in seven cases, with a survival rate of 57.1%. All patients who underwent plasmapheresis also received high‐dose/pulse steroids. In three cases, plasmapheresis was followed by IVIG, in theory allowing for a rapid benefit with subsequent long‐term effects, potentially enhancing the survival outcome. In individuals in whom standard steroid monotherapy appears to have limited benefit, there may be some additional benefit from plasmapheresis, IVIG, or a combination of both.

##### 4.1.2.3 Pyridostigmine

Pyridostigmine was initiated in three cases, specifically in patients presenting with MMM overlap, with a survival rate of 66.7%. The single patient who did not survive was treated with pyridostigmine and steroids alone, while the two survivors received a combination of pyridostigmine, plasmapheresis, and steroids. This suggests that combining pyridostigmine, plasmapheresis, and steroids may generate more favorable outcomes than pyridostigmine and steroids alone. However, this association warrants further investigation due to the limited number of cases.

##### 4.1.2.4 Mycophenolate Mofetil

Five cases were administered mycophenolate mofetil, with a survival rate of 40% and a mortality rate of 60%. It was mostly used in combination with high‐dose/pulse steroids, but in some cases, it was the sole immunomodulatory therapy. The relatively lower survival rate in these cases suggests that mycophenolate mofetil may have limited effectiveness when not used concomitantly with other treatments.

##### 4.1.2.5 Abatacept

Two cases were treated with abatacept, both of which resulted in survival. In both cases, high‐dose steroids were administered, and one patient also received mycophenolate mofetil. These positive outcomes suggest that abatacept, when combined with high‐dose steroids, may be an effective adjunctive therapy for managing irAEs.

##### 4.1.2.6 Infliximab

Infliximab was used in two cases, both of which resulted in survival. In both cases, high‐dose pulse steroids were also given. The favorable outcomes in these cases indicate that infliximab, combined with high‐dose steroids, may improve survival in patients with irAEs.

##### 4.1.2.7 Other Adjunctive Therapies

Other adjunctive therapies were used in isolated cases, including azathioprine, tocilizumab, antithymocyte, and neurohormonal antagonists. Of these, antithymocyte was associated with a single death, while the effectiveness of azathioprine, tocilizumab, and neurohormonal antagonists remains inconclusive due to the limited number of cases. Further research is needed to evaluate the role of these adjunct therapies in improving patient outcomes.

The review of these cases suggests that high‐dose steroids/pulse‐dose steroids remain a central component in the management of irAEs, particularly when combined with adjunctive therapies such as IVIG, plasmapheresis, and other immunosuppressant medications. Combining pyridostigmine with plasmapheresis and steroids appears more effective than using pyridostigmine alone. Meanwhile, mycophenolate mofetil demonstrated a lower survival rate, suggesting that it may be a less effective monotherapy. These patterns underscore the importance of combination therapy in improving patient outcomes and highlight the need for further exploration into the efficacy of different adjunctive treatments.

### 4.2 Risk Factor Insights

#### 4.2.1 Demographics and Cancer Types

The cohort predominantly consisted of older patients, with a median age of around 65 years, ranging from the mid‐30s to late 80s. Specifically, the average age of reported patients was 64 years in monotherapy utilization (62 years for females, 65 years for males) and 67 years (66 years for females, 67 years for males) for combination therapy utilization. This is consistent with the documented increased risk of cardiovascular irAEs in older patients, particularly those over the age of 65. Males accounted for 60.2% of the cases, with a notable predominance of male patients in certain cancer types, such as lung carcinoma (61.7% male) and renal cell carcinoma (80% male). This aligns with the known increased cardiovascular risk in male patients, as well as the higher susceptibility of those with these cancers to cardiovascular irAEs. Conversely, cancers like breast carcinoma and thymic carcinoma showed a female predominance, with 100% of breast cancer and 80% of thymic carcinoma cases being female.

#### 4.2.2 Treatment Modalities: Combination Versus Monotherapy

Combination immunotherapy regimens, such as nivolumab plus ipilimumab, were associated with higher mortality rates (nearly 48%). This contrasts with monotherapy with nivolumab, which had a significantly lower mortality rate (36.1%). This is consistent with documented findings that combination therapies, particularly ICI + ICI or ICI + chemotherapy, increase the risk of severe cardiovascular irAEs due to their greater immune system activation and the potential for greater toxicity. These insights reinforce the importance of carefully considering the risks and benefits of combination therapies, especially in older patients or those with preexisting cardiovascular conditions.

#### 4.2.3 Impact of Cardiac History

Patients with a history of cardiac conditions, such as hypertension and coronary artery disease, comprised 30.5% of the cohort. While the mortality rate for this group was slightly higher (38.8%) compared to those without a cardiac history (35.4%), the difference was minimal. However, the risk of cardiovascular irAEs has been documented to increase in patients with preexisting cardiac conditions, particularly with the use of combination therapies, which amplify immune system activation. Our cohort analysis was unable to demonstrate this; nonetheless, careful monitoring of patients with a history of cardiac disease is still warranted.

#### 4.2.4 Influence of Autoimmune History

Interestingly, only 4.24% of the cases had a documented autoimmune history, with conditions like Hashimoto's thyroiditis, ankylosing spondylitis, and ulcerative colitis. Despite this small sample size, the mortality rate among these patients was lower than the overall cohort, with only 20% of patients with autoimmune conditions dying from a cardiovascular irAE. This finding implies that patients with autoimmune diseases might not experience worse outcomes than the general population, at least in this specific cohort. This could suggest that patients with autoimmune conditions, who may already have baseline immune dysregulation, do not necessarily face exacerbated irAEs when treated with immune checkpoint inhibitors (ICIs).

Additionally, systemic inflammatory conditions like ulcerative colitis and psoriatic arthritis did not appear to worsen patient survival, which contrasts with expectations from some studies that suggest autoimmune patients may have an increased risk of irAEs. This observation aligns with research indicating that the presence of autoimmune diseases does not universally lead to worse cancer treatment outcomes. However, it highlights the need for further investigation with a larger sample size.

### 4.3 Summary of Outcome Insights

The review of 116 cases from 113 studies highlights critical findings regarding cardiovascular immune‐related adverse events (irAEs) during immune checkpoint inhibitor (ICI) therapy. Myocarditis was the most prevalent cardiovascular irAE, often leading to mortality. Patients receiving combination immunotherapies, such as nivolumab/ipilimumab, experienced higher mortality rates compared to those on monotherapy, indicating that the intensity of immune activation plays a key role in adverse outcomes.

Corticosteroids emerged as the primary treatment, with higher doses (≥ 1 mg/kg) being associated with improved survival. Additionally, adjunct therapies such as IVIG, plasmapheresis, and pyridostigmine were beneficial when used alongside steroids. However, mycophenolate mofetil was linked to poorer survival outcomes, suggesting limited effectiveness. Other therapies, including abatacept and infliximab, showed potential when combined with high‐dose steroids.

Older patients, particularly those aged 65 and above, were more vulnerable to cardiovascular irAEs. The data also indicated that males and patients with specific cancer types, such as lung and renal cell carcinoma, were more likely to experience these complications due to inherent cardiovascular risks within these groups.

### 4.4 Rechallenge Insights

Rechallenge after immune checkpoint inhibitor (ICI)–induced immune‐related adverse events (irAEs) was studied in 14 patients, providing insight into its feasibility and outcomes (Table 2). Most patients had lung carcinoma, while breast carcinoma, sarcoma, and renal cell carcinoma comprised smaller subsets. The patients' ages ranged from 31 to 77 years, with an even gender distribution. The irAEs typically emerged early in treatment, often within the first few cycles of therapy. Typically, irAEs were experienced between the first and sixth cycles of treatment. This suggests that if a patient is likely to experience an irAE, it would most likely occur early within the treatment window, compared to later in the treatment regimen or during the post‐treatment phase. All patients experienced Grade 3 irAEs, with cardiac complications—especially myocarditis and cardiac tamponade—being the most common initial events. Myocarditis was definitively diagnosed in five patients, underscoring its prominence as a severe immune‐related toxicity.

**TABLE 2:** Summarization of demographics, ICI drug name, cancer type, cardiovascular adverse event, treatments, timing of rechallenge, and outcome of rechallenge reported in individual case reports.

Study	Age	Gender	Drug name(s)	Type of cancer	Complication	Timing irAE (cycles)	Severity irAE	Corticosteroids	Corticosteroid type	Corticosteroid dosing (daily)	Other treatment modalities utilized	Rechallenge timing	Outcome
Airò et al. (2022) [111]	77	Female	Pembrolizumab + Axitinib	Renal Cell Carcinoma	Heart Failure	1 cycle (s/p 1st dose)	Grade 3	Yes	Unspecified	Unspecified	None	1 month (4/2 weeks respectively)	Alive
Arangalage et al. (2021) [85]	61	Female	Pembrolizumab	Melanoma	Myocarditis and Heart Failure	3 cycles	Grade 3	Yes	Methylprednisolone	1000 mg	ARB and Beta Blocker	6 months	Alive
Asai et al. (2019) [65]	62	Male	Nivolumab	Lung Carcinoma	Cardiac Tamponade	1 cycle (s/p 1st dose)	Grade 3	No	N/A	N/A	Intraperitoneal Bleomycin and Pericardiocentesis	Unknown	Alive
Gibson et al. (2016) [46]	68	Female	Nivolumab	Lung Carcinoma	Myocarditis	1 cycle (s/p 2nd dose)	Grade 3	Yes	Methylprednisolone	80 mg	Antiarrhythmic Agents	Unknown	Death
Harada et al. (2020) [142]	63	Female	Pembrolizumab	Lung Carcinoma	Cardiac Tamponade	3 cycles	Grade 3	No	N/A	N/A	Pericardiocentesis	Unknown	Death
Kumamoto et al. (2022) [125]	54	Female	Nivolumab	Hypopharyngeal cancer	Vasospastic Angina	18 cycles	Grade 3	No	N/A	N/A	Antiarrhythmic Agents	Unknown	Alive
Lee et al. (2020) [145]	31	Female	Pembrolizumab	Sarcoma	Myocarditis	13 cycles	Grade 3	Yes	Prednisone	80 mg	None	2 months	Alive
Oristrell et al. (2018) [68]	55	Female	Pembrolizumab	Breast Carcinoma	Cardiac Tamponade	6 cycles	Grade 3	Yes	Unspecified	2 mg/kg	Pericardiocentesis	Unknown	Alive
Pollock and Castillo (2023) [103]	69	Male	Pembrolizumab	Lung Carcinoma	Pericardial Effusion	12 cycles	Grade 3	No	N/A	N/A	Pericardial Window	Unknown	Alive
Saade et al. (2019) [77]	65	Male	Nivolumab	Lung Carcinoma	Cardiac Tamponade	4 cycles	Grade 3	Yes	Unspecified	Unspecified	Pericardiocentesis	16 months	Alive
Tachihara et al. (2019) [66]	70	Male	Pembrolizumab	Lung Carcinoma	Cardiac Tamponade	6 cycles	Grade 3	No	N/A	N/A	Pericardiocentesis	Unknown	Alive
Tanriverdi et al. (2020) [143]	70	Male	Pembrolizumab + Axitinib	Renal Cell Carcinoma	Heart Failure	32 cycles	Grade 3	No	N/A	N/A	Diuretics, Acetylcysteine, LMWH	Unknown	Alive
Uchida et al. (2022) [109]	69	Male	Pembrolizumab	Lung Carcinoma	Cardiac Tamponade	4 cycles	Grade 3	No	N/A	N/A	Pericardiocentesis	Unknown	Alive
Zhang et al. (2023) [105]	55	Male	Pembrolizumab + Lenvatinib	Cholangiocarcinoma	Pulmonary Arterial Hypertension	4 cycles	Grade 3	Yes	Methylprednisolone	20 mg	Mycophenolate mofetil	Unknown	Alive

Steroids played a central role in irAE management, with 7 of the 14 patients receiving corticosteroid therapy. High‐dose corticosteroids were reserved for severe conditions such as myocarditis and heart failure, while moderate doses were effective for less critical cases like pulmonary arterial hypertension. The reported dosing of high‐dose corticosteroids among the rechallenge cases includes a 1 g pulse dose of methylprednisolone or 2 mg/kg/day of corticosteroids. In comparison, moderate dosing ranged from 20 to 80 mg/day of typically methylprednisolone. Additional treatments, including antiarrhythmic agents and procedures like pericardiocentesis, were employed to address specific complications. Despite the severity of their conditions, 12 of the 14 patients (86%) survived the rechallenge, demonstrating that positive outcomes are achievable with careful selection and management. Interestingly, survival rates were similar between patients treated with steroids and those who were not, suggesting that factors beyond corticosteroid use—such as irAE timing, additional interventions, and patient health status—play a critical role in outcomes.

Among the patients who were rechallenged, two fatalities occurred: one from myocarditis despite high‐dose corticosteroid therapy and another from cardiac tamponade, not managed with corticosteroids. These cases highlight the significant risks of early and severe irAEs, reinforcing the aggressive nature of these complications and the need for timely recognition, prompt intervention, and better strategies to manage life‐threatening cardiac toxicities. The overall 86% survival rate and the absence of severe recurrence upon rechallenge suggest that resuming therapy after a period of interruption may be a viable option.

## 5 Discussion

Cardiovascular irAEs, thought to be rare adverse effects of ICI therapy, are concerning due to their variable and often insidious presentation with a high mortality rate [14]. Our systematic review distinguishes itself from prior meta‐analyses, such as those by Sharma et al. and other pharmacovigilance studies, through its granular focus on individual patient‐level data derived from case reports rather than aggregate clinical trial data or cohort data [10]. While previous large‐scale reviews have established the incidence rates of cardiovascular irAEs, our study highlights clinical nuance regarding individual management strategies and specific outcomes of rechallenge. Unique to our analysis is the detailed stratification of survival outcomes based on corticosteroid dosing regimens (high‐dose vs. low/moderate‐dose), providing real‐world evidence that aligns with, yet refines, current expert consensus. Furthermore, our study offers a dedicated analysis of ICI rechallenge safety, a clinically pivotal yet under‐reported area, demonstrating an 86% survival rate and lack of severe recurrence in this specific cohort. Finally, unlike reviews restricted solely to myocarditis, our data encompasses the full spectrum of cardiovascular toxicities, including pericardial disease, heart block, and concomitant MMM overlap syndromes, providing a more holistic view of the cardio‐immune phenotype encountered in clinical practice.

This systematic review further emphasizes that although myocarditis was the most reported cardiovascular irAE, other toxicities such as pericarditis, cardiac tamponade, acute coronary syndrome, vasospastic angina, heart block, and heart failure also have significant clinical ramifications. These manifestations may occur independently or as sequelae of underlying myocardial inflammation, underscoring the heterogeneity of ICI‐associated cardiac injury.

Across the included reports, cardiovascular irAEs frequently resulted in treatment interruption or permanent discontinuation of ICIs, underscoring their significant impact on cancer therapy continuity. Although reporting inconsistencies limited direct correlation between cardiovascular outcomes and specific ICI agents or baseline cardiovascular risk factors, several cases suggested worse outcomes among patients receiving combination therapy compared with PD‐1 monotherapy. This highlights an important clinical consideration, as the choice of ICI regimen may influence both therapeutic benefit and toxicity risk.

### 5.1 Immune Checkpoint Inhibitors

These therapies have been widely adopted for the treatment of various malignancies with encouraging results. However, they can potentially cause a myriad of complications in other organ systems. The incidence of irAEs appears to be dependent on the agent used rather than the target malignancy [9]. For pembrolizumab and ipilimumab, the development of irAEs appears to be dose dependent [15]. Although the exact mechanisms underpinning irAEs remain unclear, other inflammatory cells such as Th17 are believed to play a role, particularly for irAEs attributed to CD8 T‐cell overactivity [15].

### 5.2 Risk Factors for Cardiovascular Immune‐Related Adverse Events

Several risk factors have been documented to contribute to a higher risk of experiencing cardiovascular irAEs. These risk factors include a history of preexisting cardiovascular conditions, older age (especially over age 65), male gender, longer duration of therapy, and certain types of cancer such as lung cancer, melanoma, and renal cell carcinoma [16]. Patients with preexisting autoimmune diseases (rheumatoid arthritis, systemic lupus erythematosus, inflammatory bowel disease, etc.) are thought to be at increased risk given the baseline dysregulation of the immune system [17]. Additionally, the use of combination therapy (ICI + ICI or ICI + chemotherapy) has been documented to be the most important risk factor for cardiovascular irAEs. Combination therapy is associated with up to a twofold increased risk in some studies, with increased severity and earlier onset of cardiovascular irAEs when compared with monotherapy [16]. There is also a documented increased risk with the addition of chemotherapy to an ICI, though typically limited to agents known to be cardiotoxic (ex. anthracyclines) [18].

#### 5.2.1 Ipilimumab

Ipilimumab is a recombinant antibody against CTLA‐4. The FDA first approved its use in 2011 and is used for multiple cancers, including melanoma and non‐small‐cell lung cancer [19]. Patients do not experience common cytotoxic chemotherapy side effects, including bone marrow suppression; this is because ipilimumab works by stimulating the immune system, but this results in alternative side effects, such as irAEs, which occur in up to 90% of patients [20]. If a patient experiences a severe irAE, it is encouraged to discontinue the medication permanently; however, with minor side effects, the drug is withheld until the patient improves, and afterward, a rechallenge can be attempted [19].

#### 5.2.2 Pembrolizumab

Pembrolizumab is a humanized IgG4 antibody against PD‐1 [20]. The FDA initially approved it in 2014, and it is approved for the treatment of a variety of cancers, including lung cancer and melanoma, as well as various squamous cell cancers, lymphomas, cancers with high amounts of microsatellite instability, mismatch‐repair deficient cancers, and several others [21]. Again, like ipilimumab, irAEs are expected to occur instead of cytotoxic effects. According to the FDA, cardiovascular irAEs are rarely reported and are suggested to occur in less than 1% of patients [21]. This is compared to clinical trials and observational studies generally citing an incidence of 0.04%–1.14% [10].

#### 5.2.3 Nivolumab

Nivolumab is a fully human IgG4 antibody against PD‐1 [20]. The FDA approved its use in 2014, and it treats many cancers, including melanoma, non‐small‐cell lung cancer, and several others [22]. Similar to ipilimumab and pembrolizumab, side effects are related to immune cell overactivation. Again, cardiac complications are reported in less than 1% of patients [22].

### 5.3 Cardiac Complications

Cardiac complication is reported as a rare complication of immune checkpoint inhibitors, affecting up to 1% of patients [20, 21]. The most common adverse cardiac event that has been reported and corroborated through our analysis is myocarditis. Patients experiencing irAEs have a variety of presentations ranging from asymptomatic to severe presentations, including multiorgan failure and sudden death [23].

The proposed mechanism of cardiotoxicity is CD4+ ‐mediated T‐cell inflammation [20]. Nivolumab increases pro‐inflammatory cytokine production in CD4+ T cells, compared to cardiomyocyte‐induced apoptosis from cytotoxic medications [24]. A study reported that anti‐PD‐treated mice had more CD4+ and CD8+ T cell infiltration in the heart compared to the control group [24]. Additionally, the concept of T‐cells being the primary mediators of ICI cardiac complications is further supported by another study, which showed that mice with PD‐1 deficiency were genetically predisposed to systemic autoimmunity. The myocarditis was resultant from CD4+ and CD8+ T cells as well as antibodies against cardiomyocytes; further, mice without a PD‐1 deficiency did not develop myocarditis, which supports that prevention of myocarditis is mediated by PD‐1 [25].

#### 5.3.1 Myocarditis and Myocarditis Combined With Associated Syndromes

Myocarditis as a complication of ICI therapy typically occurs within the first 3 months after initiation, with the median onset being about a month into treatment [26]. The mechanism of ICI‐induced myocarditis is unclear, though the proposed mechanism is CD4+ ‐mediated T‐cell inflammation [20]. Nivolumab increases pro‐inflammatory cytokine production in CD4+ T cells, compared to cardiomyocyte‐induced apoptosis from cytotoxic medications [24]. Studies report that anti‐PD‐treated mice had more CD4+ and CD8+ T cell infiltration in the heart compared to the control group [24]. Additionally, the concept of T‐cells being the primary mediators of ICI irAE is further supported by another study, which showed that mice with PD‐1 deficiency were genetically predisposed to systemic autoimmunity [25]. The myocarditis resulted from CD4+ and CD8+ T cells as well as antibodies against cardiomyocytes; further, mice without a PD‐1 deficiency did not develop myocarditis, which supports that prevention of myocarditis is mediated by PD‐1 [25].

#### 5.3.2 MMM Overlap Syndrome

Although most cases of myocarditis from ICI therapy occur in isolation, concurrent development of myositis and/or myasthenia gravis has been seen in up to 30% of cases [27]. The underlying mechanism is unclear, though it is proposed that it is similar to isolated myocarditis with evidence of molecular mimicry within the PD‐1 signaling pathways. Due to this signaling dysfunction, the body is unable to regulate the autoimmune response [28]. Given the rates of co‐occurrence of these three adverse effects, when one of the three overlapping adverse effects is present, it is essential to screen patients for the other two. Further investigation into this overlap syndrome is needed to develop an optimal approach to diagnosis and management.

#### 5.3.3 Pericardial Disease

Pericardial disease occurs in approximately 0.3% of patients receiving ICI therapy and is associated with a mortality rate of 13% to 21% and is seen, on average, 6 months after initiating therapy [20, 29]. The presentation of these conditions is usually nonspecific, with shortness of breath being the prevailing symptom. Patients frequently show EKG changes during pericardial disease [20]. If the pericardial fluid is analyzed, it will show the absence of malignancy cells and the presence of lymphocytes and plasma cells [23]. Interestingly, treatment with anti‐PD‐1 or anti‐PD‐L1 therapy is associated with a higher risk of pericardial disease when compared to anti‐CTLA‐4 therapy [30].

#### 5.3.4 Heart Block and Arrhythmias

Immune checkpoint inhibitor therapy is associated with several reported arrhythmias, including atrial fibrillation, ventricular tachycardia or fibrillation, and atrioventricular conduction disorders [31]. These may occur with or without myocarditis. It is unknown what causes these arrhythmias, but it is hypothesized that local inflammation or fibrosis may play a role.

#### 5.3.5 Heart Failure

The mechanism of heart failure related to ICI is not clear. As previously highlighted, proposed mechanisms include CD4+ ‐mediated T‐cell inflammation [20]. PD‐L1 signaling may have a cardioprotective activity that suppresses excessive myocardial inflammation. Therefore, inhibition of PD‐1 or PD‐L1 can result in autoimmune T‐cell‐mediated cardiotoxicity. Direct inhibition of PD‐L 1 may accelerate pre‐existing heart disease and cause non‐inflammatory cardiomyocyte dysfunction [32].

#### 5.3.6 Acute Coronary Syndrome (ACS) and Vasospastic Angina

The mechanism of this cardiac complication is unknown. However, there are hypotheses that it may be due to ruptured atherosclerotic plaques, coronary spasms, or coronary vasculitis due to the direct activation of T cells [31]. Additionally, ACS may also be a result of ICI‐induced arrhythmias [20].

#### 5.3.7 Other Cardiovascular irAEs

Emerging evidence suggests that immune checkpoint inhibitors may affect coronary vasculature by influencing vascular inflammation and atherogenesis. Mechanistically, the PD‐1/PD‐L1 axis appears to play a protective role; in mouse models, PD‐L1/2 deficiency has been shown to result in significantly increased atherosclerotic burden and enhanced infiltration of CD4+ and CD8+ T cells into the aorta, driven by dysregulated T‐cell activation by antigen‐presenting cells [33].

This pro‐inflammatory mechanism is further supported by recent positron emission tomography (PET)‐based studies in humans [34]. In a study of 20 patients with known melanoma treated with ICI therapy, ICI use was associated with significant inflammatory activity in large arteries, including the aorta. This suggests that ICI therapy may act as a critical moderator of atherosclerosis, potentially increasing the long‐term risk of cardiovascular events even in younger populations.

The clinical translation of these mechanisms is becoming increasingly apparent in large‐scale safety analyses. A meta‐analysis of randomized clinical trials found that ICI use was associated with a threefold increase in the risk of atherosclerotic cardiovascular events, including myocardial infarction and cerebral arterial ischemia, alongside increased risks for venous thromboembolism [35].

At the cellular level, the interplay between metabolic reprogramming and immune signaling is pivotal. Targeted deletion of PD‐1 in myeloid cells has been shown to induce metabolic changes, including elevated cholesterol, which is required for the differentiation of inflammatory macrophages. While this mechanism is key for antitumor immunity, it underscores the systemic metabolic and vascular trade‐offs potentially inherent to PD‐1 blockade [36].

### 5.4 Clinical Perspectives

#### 5.4.1 Diagnosis of ICI irAEs

Given the myriad presentations of ICI irAEs, a high index of clinical suspicion is the key to early diagnosis. Given the high potential mortality and morbidity associated with these conditions, hospitalization would be a prudent initial step. Standard hematologic investigations should include a complete hemogram, liver and renal function tests, and assessment of inflammatory markers such as an erythrocyte sedimentation rate or C‐reactive protein level. Several reports recommend establishing a cardiac baseline with cardiac biomarkers, including serum creatine kinase, N‐Terminal Pro‐B‐Type Natriuretic Peptide (NT‐proBNP), and troponin levels. Electrocardiograms (EKGs), echocardiography, or cardiac magnetic resonance imaging (cMRI) may also be warranted. This becomes especially important in patients with pre‐existing heart disease and baseline laboratory abnormalities [10].

#### 5.4.2 ICI Cardiac Complication Management

Management of cardiovascular irAEs is guided by recommendations from the American Society of Clinical Oncology (ASCO) and the National Comprehensive Cancer Network (NCCN), both of which emphasize immediate discontinuation of immunotherapy, early initiation of high‐dose corticosteroids, and multidisciplinary evaluation [37]. These guidelines recommend starting intravenous methylprednisolone at 1–2 mg/kg/day (or 1 g/day for fulminant cases), with a gradual oral taper once clinical and biomarker improvement occurs. Additional diagnostic steps, such as cardiology consultation, cardiac MRI, and, when appropriate, endomyocardial biopsy, are advised to confirm the diagnosis and assess severity. For patients who do not improve within 24–48 h, escalation to second‐line immunosuppressive therapy (e.g., mycophenolate mofetil, IVIG, anti‐thymocyte globulin, or abatacept) is recommended, while infliximab is avoided due to concerns for worsening heart failure. Rechallenge is generally discouraged after grade ≥ 2 myocarditis and should only be considered after multidisciplinary review.

These guideline principles provide a framework for clinical decision making and align with current literature describing the presentation, grading and management of irAE cardiotoxicities. ICI‐related cardiovascular irAEs are graded by severity from grade 1 (asymptomatic patients with abnormal biomarkers or electrocardiographic changes) to grade 4 (severe or life‐threatening disease with echocardiographic demonstration of a left ventricular ejection fraction < 50% or a cMRI diagnostic for myocarditis with cardiac biomarkers > 3 times the upper limit of normal) [7, 38]. Even the most benign symptom reported may indicate an underlying developing cardiac complication. ICIs should promptly be discontinued when an irAE is suspected, and treatment should be initiated. Although there is a dearth of evidence‐based guidelines to treat ICI‐related irAEs, current literature supports the use of steroid therapy with higher doses (initial dose > 2 mg/kg) appearing more likely to have favorable outcomes than low dose regimens (initial dose < 1 mg/kg) [26]. Some studies showed that patients who received corticosteroids within 24 h of presentation, regardless of dosage, showed the best outcome compared to those who received corticosteroids after 72 h [39]. This may be attributed to the fact that ICI‐associated myocarditis often overlaps with other cardiovascular disorders, including acute coronary syndrome and congestive heart failure, and confirming the diagnosis of ICI myocarditis often takes more than 24 h in real life [40, 41].

Thus, it is recommended to start steroids while pursuing the workup. If an alternative clinical syndrome becomes the more compelling diagnosis, then steroids can be stopped. Once the diagnostic workup reveals definite or probable myocarditis, steroids should be continued and tapered over at least 4–6 weeks, depending on the resolution of cardiac biomarker elevation and normalization of cardiac function. Although myocarditis may worsen initially following the initiation of steroids, this is observed less frequently with higher doses [7]. Absence of response within the first 24–48 h should prompt consideration of alternative immunomodulatory therapies, including plasmapheresis, IVIG, anti‐thymocyte globulin, mycophenolate, tacrolimus, or infliximab. Abatacept, a CTLA‐4 agonist, may be useful in steroid‐refractory cases [42]. In some instances, it may be reasonable to attempt to re‐challenge with the suspected culprit ICI, as current literature supports that it may be tolerated without recurrence of the irAE. However, this would be inadvisable in patients with reactions more severe than grade 1 [7]. Other treatments addressing symptom management are often used, including GDMT in heart failure, antiarrhythmics, pacemaker implantation for heart block, pericardiocentesis, or pericardial windows in cardiac tamponade.

**FIGURE 10:**
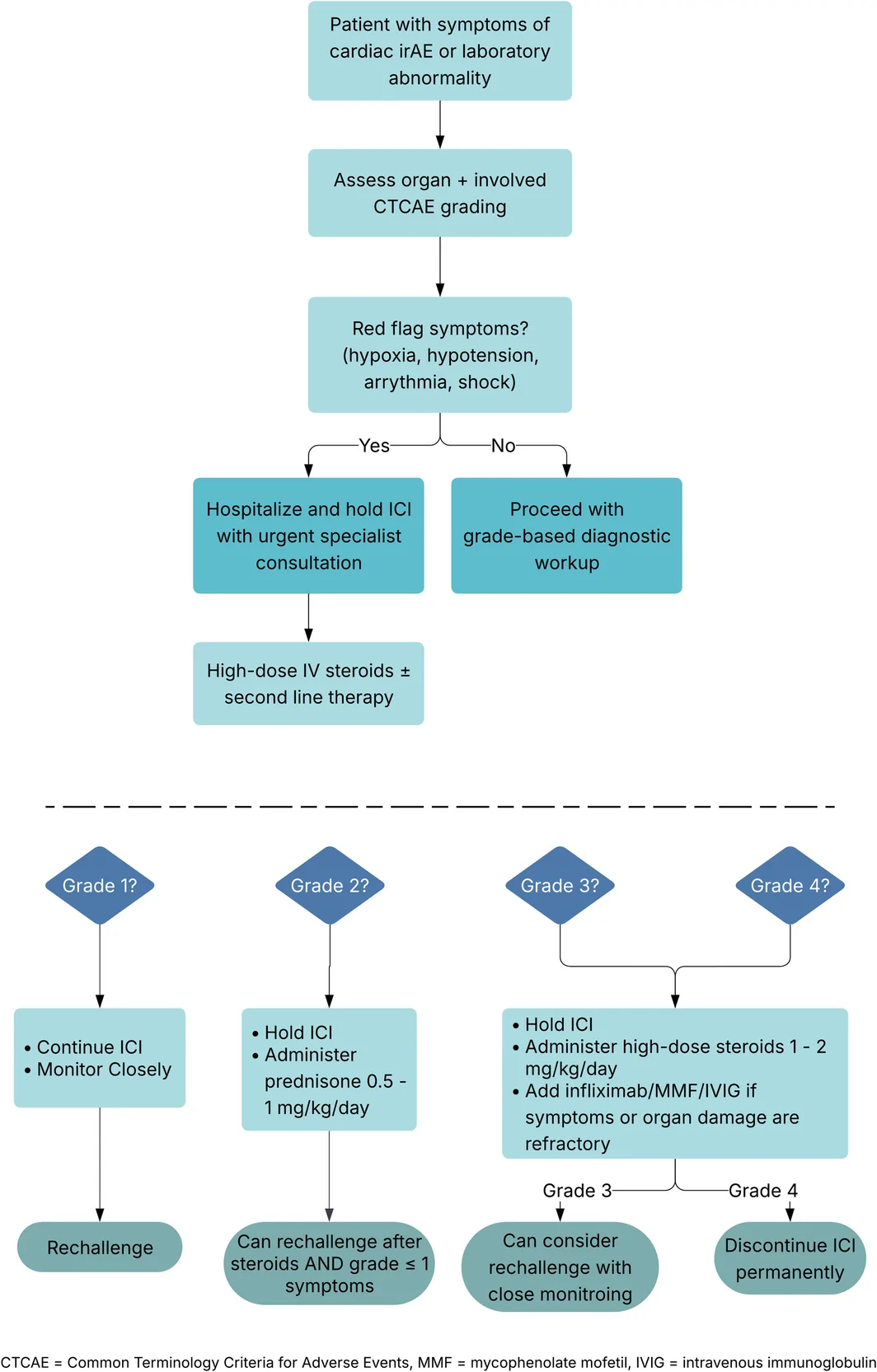
Grade‐based management algorithm for immune checkpoint inhibitor‐associated cardiovascular irAEs.

Several studies have recommended that ICI therapy be discontinued even after minor irAEs, at least temporarily, and if significant irAEs occur, permanently. Due to the long half‐life of ICI therapies, steroid and adjuvant treatment often need to be tapered over several weeks after the discontinuation of ICI to ensure the cardiac complication does not worsen or persist [20]. A proposed diagnostic and management algorithm summarizing these ASCO and NCCN guideline principles with the current literature is presented in Figure 10.

#### 5.4.3 Surveillance of Cardiovascular irAEs

Although cardiovascular irAEs occur infrequently relative to the overall use of immune checkpoint inhibitors, early detection is critical given their disproportionately high morbidity and mortality; however, screening and surveillance strategies for outpatient monitoring are not well defined. The best surveillance approach would be based on clinical risk assessment, followed by biomarker and imaging data. The risk factors for ICI myocarditis remain to be clarified, but the most validated risk factor thus far is combination ICI therapy (e.g., anti–CTLA‐4 plus anti–PD‐1) [43]. Currently, there are ongoing international efforts to better understand the risk factors and the full clinical spectrum of ICI myocarditis [44]. The most highlighted cases in the literature are the most severe cases of ICI myocarditis, and these patients often present early after initial exposure (within the first 1 to 2 months). Many programs use a symptom‐based approach in patients receiving ICI without relying on routine troponin, BNP, or echocardiogram monitoring. Some studies show that no overt or subclinical myocarditis was detected by monitoring ECG and troponin levels in all patients treated with combination ICI therapy [43]. Baseline assessment of cardiac troponins and natriuretic peptides has been recommended by the 2022 European Society of Cardiology (ESC) guideline on cardio‐oncology guidance as part of cardiovascular risk stratification for patients scheduled to receive ICI, with the aim of identifying those who may be predisposed to subclinical myocardial injury and could benefit from closer surveillance. However, direct evidence validating the prognostic value of these biomarkers specifically in ICI‐treated populations remains limited, and current recommendations are therefore based primarily on expert consensus. This lack of definitive guidelines highlights the need for a multidisciplinary team approach, including the involvement of the patients' primary care physicians, oncologists, and cardiologists for appropriate patient care and timely intervention if warranted.

## 6 Limitations

The limitations of this study are largely linked to its reliance on case reports. The use of case reports subjects this study to a potential selection bias of unusual and unique cardiac complications associated with ICI therapy. An additional limitation is that the literature search was completed on April 1, 2024. Given the rapidly evolving literature on cardio‐oncology and ICI‐related cardiovascular toxicities, relevant case reports, case series, guidelines, consensus statements, and other publications released after this date may not be represented in this review. Additionally, publication bias from the authors reporting the cases is possible. As a result of these potential biases, the true prevalence and management of cardiac complications associated with ICI therapy are difficult to determine from an analysis of reported cases. There were few case reports with cardiac complications associated with the use of other ICI therapies; as these agents become more widely used, their association with cardiovascular irAEs will need to be further evaluated. It is also difficult to establish causal relationships between ICI use and cardiac complications. However, despite these limitations, using case reports is fundamental in detecting patterns, generating hypotheses, and promoting the need for further research in these areas.

## 7 Conclusion

Immune checkpoint inhibitor therapy has been associated with several cardiac complications, including the most reported myocarditis and pericarditis, but also heart failure, cardiac tamponade, heart block and arrhythmias, Takotsubo cardiomyopathy and even ACS. Although cardiac complications are rare events associated with ICI therapy, they are concerning owing to their high mortality rate. This systematic review and meta‐summary provide a better understanding of the risk factors, clinical presentation, and treatment of cardiovascular complications of ICI therapy. The efficacy and cost‐effectiveness of baseline screening and routine surveillance for cardiovascular toxicity remain to be determined. This meta‐summary highlights the significant risks of severe cardiovascular irAEs, reiterating the need for timely recognition, prompt intervention, and more standardized approaches for management. The overall 86% survival rate and the absence of severe recurrence upon rechallenge suggest that resuming therapy after a period of interruption may be a viable option. However, more studies are needed to definitively answer the important question of ICI rechallenge.

## Author Contributions

**Shea‐Lee Godin:** conceptualization, methodology, validation, investigation, writing – original draft, writing – review and editing, supervision. **Tyler Kingma:** methodology, validation, investigation, writing – original draft, writing – review and editing. **Ashwin Pillai:** validation, writing – review and editing. **Obada Kholoki:** visualization, writing – review and editing. **Agnes S. Kim:** writing – review and editing, supervision.

## Funding

The authors have nothing to report.

## Disclosure

Protocol can be accessed through contacting the corresponding author.

## Ethics Statement

This article contains no studies with human participants or animals performed by authors.

## Conflicts of Interest

The authors declare no conflicts of interest.

## Data Availability

The data that support the findings of this study are available from the corresponding author upon reasonable request.
